# Sero-prevalence and determinants of Hepatitis B among a cohort of HIV-infected women of reproductive age in Nigeria

**DOI:** 10.1371/journal.pone.0236456

**Published:** 2020-09-17

**Authors:** Olusegun Adewale Adeyemi, Ijeoma Uchenna Itanyi, Chamberline Ekene Ozigbu, Nicole Stadnick, Kiyomi Tsuyuki, Olanrewaju Olayiwola, Amaka Grace Ogidi, Chuka Eze, Gregory Alan Aarons, Chima Ariel Onoka, Echezona Edozie Ezeanolue

**Affiliations:** 1 International Research Center of Excellence, Institute of Human Virology Nigeria, Abuja, Nigeria; 2 Department of Epidemiology and Public Health, Graduate Program In Life Sciences, University of Maryland, School of Medicine, Baltimore, MD, United States of America; 3 Center for Translation and Implementation Research, University of Nigeria Nsukka, Enugu, Nigeria; 4 Department of Community Medicine, University of Nigeria Nsukka, Enugu, Nigeria; 5 Department of Health Services Policy and Management, University of South Carolina, Columbia, South Carolina, United States of America; 6 Department of Psychiatry, University of California, San Diego, ‎California, United States of America; 7 UC San Diego Dissemination and Implementation Science Center, San Diego, California, United States of America; 8 Division of Infectious Diseases & Global Public Health, Department of Medicine, University of California, San Diego, California, United States of America; 9 Caritas Nigeria, Abuja, Federal Capital Territory, Nigeria; 10 Vitira Health, Arlington, Virginia United States of America; 11 Healthy Sunrise Foundation, Las Vegas, Nevada, United States of America; University of Cincinnati College of Medicine, UNITED STATES

## Abstract

**Introduction:**

Sub-Saharan Africa houses over two-thirds of the 37 million people living with human immunodeficiency virus (HIV) globally and of this, 5–20% are co-infected with Hepatitis B virus (HBV). This is double jeopardy, especially for women of reproductive age in these settings, who can transmit both viruses vertically as well as horizontally to their children. The objectives of this study were to investigate the prevalence and determinants of HBV among women of reproductive age living with HIV.

**Methods:**

This was a cross-sectional study of HIV-infected women of reproductive age in Benue State, Nigeria. Participants were eligible for the study if they were HIV-infected women (ages 18–45 years) receiving care from any of the selected study sites. A global rapid hepatitis B surface antigen (HBsAg) antibody test strip was used to test for HBsAg in plasma. A pretested questionnaire was used to collect data on sociodemographic, clinical and lifestyle characteristics of participants. We estimated prevalence of HBV infection and used multivariable logistic regression to determine factors associated with the infection at a significance level of <0.05.

**Results:**

A total of 6577 women were screened for HBsAg. The prevalence of HBV was 10.3% (95% CI: 9.5–10.9%). Age, parity and male partner’s HIV status were found to be associated with having HBV infection. Compared to women older than 40 years, the odds of HBV infection increased significantly with increasing age until age 35 years and decreased significantly with increasing parity (versus no parity). Women with HIV-infected partners and those without a partner had higher odds of HBV infection compared to women with HIV-negative partners.

**Conclusion:**

HBV is hyperendemic among HIV-infected women of reproductive age in North Central Nigeria. Specific programs targeting HBV testing, vaccination and treatment of all women of reproductive age need to be developed in this resource-limited, high-need setting.

## Introduction

Viral hepatitis caused 1.34 million deaths in 2015 and this is increasing over time [1]. In 2016, the World Health Organization (WHO) declared Hepatitis B virus (HBV) infection a global emergency [2], following the infection of 257 million people and the death of almost one million people, all mostly from Sub-Saharan Africa [1]. As antiretroviral therapy reduced the morbidity and mortality associated with Human Immunodeficiency Virus (HIV) globally, it also unmasked the chronic effect of HBV on HBV-HIV co-infected patients [3]. For example, there has been a rise in the incidence of end-stage liver diseases (ESLD) among HBV-HIV co-infected patients [4].

Sub-Saharan Africa houses over two-thirds of the 37 million people living with HIV globally and 5–25% of them are co-infected with HBV-HIV [5]. This is double jeopardy for women of reproductive age in these settings, who can transmit both viruses vertically (via childbirth) and horizontally (via saliva or open wounds) to their children. Mother to child transmission of HBV is a common cause of chronic HBV infection in Sub-Saharan Africa [6, 7]. As a result, it is critical to prevent HBV and HIV transmission among women of reproductive age, to protect women as well as the future generation.

Previous studies on HBV among women of reproductive age in Africa have shown that the prevalence ranges mostly from high to moderate endemicity in most regions. Hepatitis B Surface Antigen (HBsAg) or HBV prevalence of 7% or higher is classified as highly HBV endemic [7, 8]. In Eastern Africa, a cross-sectional study reported HBV prevalence of 8% [9]. Two southern African countries, Botswana and South Africa reported HBV prevalence of 4%, and 10% [10], respectively. In Central Africa, it was reported that the HBV prevalence was 4% [11]. HBV prevalence among women of reproductive age in Nigeria varies depending on the context of study settings, ranging from 7–12% in studies conducted across the country [12–18]. Overall, there is a paucity of HBV prevalence studies among women of reproductive age in West Africa, and inconsistent HBV prevalence reports in Nigerian studies.

Despite extensive research on the prevalence of HBV among women of reproductive age in Nigeria, very few studies have examined the HBV prevalence among HIV-infected women. Furthermore, most HBV prevalence studies in Nigeria and West Africa had small sample sizes as part of their study limitations. According to the most recent Nigeria HIV/AIDS indicator and impact survey [19], there is an epidemic geographical shift of the HIV burden, moving the epicenter from the North central to the North Eastern and South South region of the country. Given that HIV and HBV have shared routes of transmission, the HBV prevalence, especially among HIV-infected persons might be impacted by this shift. The primary objective of this study is to report the prevalence and identify health, lifestyle, and sociodemographic determinants of HBV co-infection among women living with HIV in a large sample cohort from North Central Nigeria. The primary hypothesis was that the odds of HBV co-infection will be highest among the least educated, youngest age group (18–25 years) and multiparous women in this study. This hypothesis was developed based on previous studies [11, 20, 21], that have reported that younger age, lower level of education and greater number of children (parity) were associated with higher odds of HBV infection among women of reproductive age in Sub-Saharan Africa. This study uniquely contributes to and extends upon existing HIV research in sub-Saharan Africa by more clearly identifying the association between HIV and HBV co-infection in a resource-limited setting and providing insight into targeted efforts to develop and implement public health interventions to curb the HIV-HBV co-infection syndemic.

## Materials and methods

### Study context, design and participants

The current study leveraged a scale-up of the integrated mhealth intervention to prospectively study a cohort of HIV-infected women of reproductive age receiving care at five comprehensive HIV treatment centers in Benue State, Nigeria. The development of the integrated mHealth platform is described in a previous study [22]. In brief, the mHealth platform is capable of storing encrypted patient information on a patient-held smartcard and smart phone mobile application. This solution is linked with a secure web-based engagement management database. Additional capabilities include the ability to view patient records on a mobile app without an internet connection; this aids in healthcare decision-making at the point of care in low resource environments [23]. Benue State has an estimated total population of 5,138,531, of whom 49.6% are female. Most of the population resides in the rural areas and farming is the predominant occupation in the state (in 74.8% of the population) [24]. About 70% of the female population has less than a secondary school education with a literacy rate of 52.8% [25].

Study sites were selected using the following criteria: a comprehensive HIV treatment facility, received funding support from US President’s Emergency Plan for AIDS Relief (PEPFAR) through Caritas Nigeria, had records of a high volume of HIV-infected women (≥ 2000 women on treatment), and offered free HIV testing services, antiretroviral therapy for both adults and children, and services for prevention of MTCT. There were 21 comprehensive HIV treatment facilities in Benue State, seven had ≥ 2000 women on treatment, out of which five had administrative heads who gave approval for participation in the study and were selected. The five selected health facilities were NKST Hospital, Zaki Biam in Ukum Local Government Area (LGA), St. Vincents Hospital, Aliade in Gwer East LGA, St. Monica's Hospital, Adikpo in Kwande LGA, Bishop Murray Medical Center, Makurdi in Makurdi LGA, and St. Thomas Hospital, Ihugh in Vandeikya LGA.

HIV-infected women were eligible to participate in this study if they were of reproductive age (18–45 years) and received ART from any of the five selected treatment sites.

### Data collection

Trained health workers offered pre-printed mhealth smartcards with unique patient identifiers to eligible HIV-infected women as they presented for their routine pre-scheduled clinic appointments. The study’s purpose and procedures were explained to these women, and written informed consent was obtained from those who chose to participate in the study.

### Laboratory testing

The research staff who had been trained in phlebotomy and standard laboratory practices collected venous blood from the participants using a vacutainer needle and an EDTA bottle.

Global rapid HBsAg antibody test strips manufactured by Model Laboratories, USA, and validated and approved by the National Agency for Food and Drug Administration and Control, Nigeria (NAFDAC Reg No: 03–1192) [26], were used to test for hepatitis B surface antigen in plasma. This test has a relative sensitivity of >99.9% and a relative specificity of 99.4%. During testing, the plasma specimen migrates upwards on the membrane of the test strip to react with the anti-HBsAg antibody on the test region of the strip to produce a coloured line. Presence of two distinct coloured lines on the test strip denotes a positive test result. The result of the test was read 15 minutes after application of the blood sample to the test strip. The test results were shared with the women and their health provider as they was no need for referral since they were already enrolled in care. To ensure quality control, the rapid test strips were validated by comparing to known positive and negative tests which had been confirmed through ELISA.

### Questionnaire administration

Trained research assistants used pretested semi-structured questionnaires to collect data on sociodemographic and clinical characteristics, and lifestyle habits of the study participants. Sociodemographic characteristics included age, marital status, highest level of education, occupation, number of living children, and partner’s age. Clinical characteristics included viral load, ART start date, current ART regimen, partner’s HIV status and whether participant was currently pregnant. Participants were also asked about their tobacco smoking and alcohol habits. This study was carried out between June and December, 2017.

Ethical approval for this study was obtained from the Health Research Ethics Committee of the University of Nigeria Teaching Hospital (NHREC/05/01/2008B-FWA00002458-1RB00002323). Approval was obtained from the health administrators of all the study sites and written informed consent was obtained from participants.

### Statistical analysis plan

#### Analytical plan for Aim 1: To determine the prevalence of HBV co-infection

The primary dependent variable was prevalent HBV infection, categorized as positive or negative. The prevalence of HBV co-infection in this study was assessed by the use of proportions and 95% confidence intervals.

#### Analytical plan for Aim 2: To determine factors associated with HBV co-infection

To determine association with prevalent HBV infection, the independent variables assessed were sociodemographic characteristics (age, marital status, highest level of education, occupation, number of living children, and partner’s age), clinical characteristics (viral load, duration on ART [defined as date of last clinic visit minus date of starting ART], current ART regimen, partner’s HIV status and current pregnancy) and lifestyle habits (tobacco smoking and alcohol habits) of participants.

Categorical variables were assessed by frequencies and percentages with 95% confidence interval (CIs), and continuous variables were assessed with medians and interquartile ranges. We compared distributions of categorical variables by HBV status with Pearson’s chi-squared and Fisher’s exact tests as appropriate. Bivariate and multivariable logistic regression models estimated odds ratios (ORs) and 95% confidence intervals (CIs) of factors associated with HBV. For the multivariable regression models, we included variables known to be associated with HBV infection based on literature review (age, parity, level of education and ART regimen), biological plausible variables (current ART regimen and marital status) as well as factors associated with HBV infection in this study at a significance level of ≤ 0.20 in the bivariate analysis [27]. Criteria for retention in the multivariable model included a significant likelihood ratio test from comparing the full model with the partial model (without the variable of interest). Associations with a p-value of less than 0.05 were considered statistically significant. Within ordered categorical data, we assessed p-value for trend using the Cochran-Armitage test for trends [28].

Logistic regression was used to test the hypothesis that age, parity and highest educational level of participants would be associated with HBV infection. Statistically significant associations were probed with a Wald chi-square test and 95% confidence interval estimation of adjusted odds ratio.

For missingness, we used a pairwise deletion approach (excluded variables without study outcomes), and multiple imputation. Fraction of missing information [25] (FMI) for continuous variables and types of missingness [29] were reported. Sensitivity analysis comparing outcomes from pairwise deletion and multiple imputation methods of handling missing data were conducted. Data analysis was performed using SAS version 9.4 (SAS Institute, Cary, NC USA) and Stata version 11 (College Station, TX: StataCorp LP).

## Results

Of the 8825 women of reproductive age who participated in the patient-held smartcard scale-up intervention, 75% (6577/8825) had Hepatitis B surface antigen laboratory test done during the study period. We report the results of the women who were tested for HBsAg. Socio-demographic characteristics of the women are shown in Table 1. The majority (92.3%) of participants were on 1^st^-line antiretroviral drugs, with TDF/3TC/EFV (Tenofovir Disoproxil Fumarate / Lamivudine/ Efavirenz) being the most common regimen. Of the 6419 women with complete data on current ART regimen, 4320 (67.3%) were on a Tenofovir-based regimen. The median duration on ART was 4.5 years (Inter Quartile Range [IQR]: 2.6 to 6.5 years). About 35.2% of the women had male partners who were HIV-infected (Table 1).

**Table 1:** Sociodemographic and clinical characteristics of female Hep B testers who participated in a patient-held smartcard scale-up study in Benue State, Nigeria (N = 6,577).

Variable	Frequency (N), (%)
**Age (Years)**	
***Median (IQR)*: *33 (28–37)***	
18–25	800 (12.16)
26–30	1531 (23.28)
31–35	1938 (29.47)
36–40	1316 (20.01)
>40	885 (13.46)
Missing	107 (1.63)
**Marital status**	
Single	642 (9.76)
Married	3768 (57.29)
Widowed	725 (11.02)
Divorced/Other	474 (7.20)
Missing	968 (14.72)
**Level of education**	
No formal education	1745 (26.53)
Primary	2715 (41.28)
Secondary or higher	2074 (31.53)
Missing	43 (0.65)
**Occupation**	
Farmer	4411 (67.07)
Trader	1247 (18.96)
Other	594 (9.03)
Unemployed	173 (2.63)
Missing	152 (2.31)
**Parity**	
***Median (Range)*: *3 (0–12) children***	
None	875 (13.30)
1–2	2312 (35.15)
3–4	2011 (30.58)
5 or more	1367 (20.78)
Missing	12 (0.18)
**Partner’s age (Years)**	
15–35	1264 (19.22)
36–45	2024 (30.77)
46–55	1072 (16.30)
>55	528 (8.03)
Missing	1689 (25.68)
**Partner’s HIV status**	
Negative	2170 (32.99)
Positive	2317 (35.23)
Unknown	517 (7.86)
No partner	1527 (23.22)
Missing	46 (0.70)
**Current ART regimen**	
TDF/3TC/EFV	4130 (62.79)
AZT/3TC/NVP	1943 (29.54)
Other	346 (5.26)
Missing	158 (2.40)
**Duration on ART (Years)**	
***Median (IQR)*: *4*.*5 (2*.*6–6*.*5)***	
< 1	352 (5.35)
1–3	1642 (24.97)
4–6	2595 (39.46)
> 6	1850 (28.13)
Missing	138 (2.10)
**Currently pregnant**	
No	6201 (94.28)
Yes	323 (4.91)
Missing	53 (0.81)
**HBV status**	
Negative	5903 (89.80)
Positive	674 (10.25)

*Percentages may not sum up to 100%.

The prevalence of Hepatitis B virus was 10.3% (95% CI: 9.5–10.9%) among our sample of HIV-infected women.

In bivariate analyses (Table 2), significant differences were seen in participants’ age, partner’s HIV status, and parity between HBV positive and negative women. Women who tested HBV positive were significantly younger, had less children and were more likely to have a HIV positive partner compared with HBV negative women.

**Table 2:** Bivariable analysis of socio-demographic and clinical characteristics associated with HBV infection among female HBV testers and participants of patient-held smartcard scale-up study in Benue State, Nigeria.

HBV (N = 6,577)				
	Negative (N = 5,903)	Positive (N = 674)	Total	P-Value
**Age (years) *Median (IQR)***	***33 (28–38)***	***32 (28–36)***		
18–25	720 (12.39)	80 (12.12)	800	**0.038**
26–30	1359 (23.39)	172 (26.06)	1,531	
31–35	1727 (29.72)	211 (31.97)	1,938	
36–40	1185 (20.40)	131 (19.85)	1,316	
>40	819 (14.10)	66 (10.00)	885	
**Marital status**				
Married	3389 (67.30)	379 (66.14)	3,768	0.475
Single	569 (11.30)	73 (12.74)	642	
Widowed	658 (13.07)	67 (11.69)	725	
Divorced/Other	420 (8.34)	54 (9.42)	474	
**Level of education**				
None	1582 (26.98)	163 (24.81)	1,745	0.293
Primary	2422 (41.31)	293 (43.07)	2,715	
Secondary or higher	1859 (31.71)	215 (32.12)	2,074	
**Occupation**				
Farmer	3954 (68.56)	457 (69.45)	4,411	0.926
Trader	1125 (19.51)	122 (18.54)	1,247	
Other	534 (9.26)	60 (9.12)	594	
Unemployed	154 (2.67)	19 (2.89)	173	
**Parity**				
None	763 (12.94)	112 (16.72)	875	**0.016**
1–2	2067 (35.06)	245 (36.57)	2,312	
3–4	1820 (30.87)	191 (28.51)	2,011	
>4	1245 (21.12)	122 (18.21)	1,367	
**Partner’s HIV status**				
Positive	2049 (34.97)	268 (39.88)	2,317	**0.008**
Negative	1985 (33.88)	185 (27.53)	2,170	
Unknown	463 (7.90)	54 (8.04)	517	
No partner	1362 (23.25)	165 (24.55)	1,527	
**Current ART regimen**				
TDF/3TC/EFV	3697 (64.06)	433 (66.82)	4,130	0.216
AZT/3TC/NVP	1755 (30.41)	188 (29.01)	1,943	
Other	319 (5.53)	27 (4.17)	346	
**Currently Pregnant**				
No	5570 (95.08)	631 (94.74)	6,201	0.702
Yes	288 (4.92)	35 (5.26)	323	
**Duration on ART(Years)**				
< 1	311 (5.37)	41 (6.30)	352	0.780
1–3	1474 (25.47)	168 (25.81)	1,642	
3–6	2336 (40.36)	259 (39.78)	2,595	
>6	1667 (28.80)	183 (28.11)	1,850	

Six variables met the criteria (literature review, biologic plausibility and bivariate analysis association with HBV) for inclusion in the multivariable model (Table 3).

**Table 3:** Multivariable analysis of factors associated with HBV infection in female participants of patient-held smartcard scale-up study in Benue State, Nigeria (N = 6,531).

	Unadjusted OR (95% CI)	Adjusted OR* (95% CI)
**Age (Years)**		
18–25	1.38 (0.98–1.94)	**1.56 (1.03–2.37)**
26–30	**1.57 (1.17**–**2.11)**	**1.74 (1.20**–**2.50)**
31–35	**1.52 (1.13**–**2.02)**	**1.69 (1.20**–**2.39)**
36–40	**1.37 (1.01**–**1.87)**	**1.56 (1.10**–**2.24)**
>40	Reference	Reference
**Marital Status**		
Married	0.87 (0.69–1.14)	1.02 (0.75–1.38)
Widowed	0.79 (0.56–1.13)	0.96 (0.65–1.43)
Divorced/Others	1.00 (0.69–1.46)	1.11 (0.75–1.66)
Single	Reference	Reference
**Level of education**		
None	Reference	Reference
Primary	1.17 (0.96–1.43)	1.02 (0.81–1.29)
Secondary or higher	1.12 (0.91–1.40)	0.90 (0.70–1.16)
**Occupation**		
Farmer	0.94 (0.58–1.52)	
Trader	0.88 (0.53–1.47)	
Others	0.91 (0.53–1.57)	
Unemployed	Reference	
**Parity**		
None	Reference	Reference
1–2	0.80 (0.64–1.03)	0.81 (0.62–1.06)
3–4	**0.72 (0.56**–**0.92)**	**0.68 (0.50**–**0.92)**
>4	**0.67 (0.51**–**0.88)**	0.73 (0.52–1.02**)**
**Partner’s HIV status**		
Negative	Reference	Reference
Positive	**1.40 (1.15**–**1.71)**	**1.53 (1.22**–**1.91)**
Unknown	1.25 (0.91–1.72)	1.30 (0.92–1.86)
No Partner	**1.30 (1.04**–**1.62)**	**1.46 (1.13**–**1.89)**
**Current ART Regimen**		
TDF/3TC/EFV	Reference	Reference
AZT/3TC/NVP	0.92 (0.76–1.10)	0.91 (0.75–1.10)
Others	0.72 (0.48–1.08)	0.66 (0.42–1.04)
**Duration on ART(Years)**		
< 1	Reference	
1–3	0.87 (0.60–1.24)	
3–6	0.84 (0.60–1.19)	
>6	0.83 (0.58–1.19)	
**Currently Pregnant**		
No	Reference	
Yes	1.07(0.75–1.54)	

Logistic regression was used to estimate odds ratios and 95% confidence intervals.

**Bolded** indicates significance at p-value<0.05.

*The final model was adjusted for age, education level, parity, partner’s HIV status and ART regimen.

Compared with women older than 40 years, those within 26–30 years had the highest odds of HBV infection, a 74% increase in odds (AOR: 1.74, 95% CI [1.20, 2.50]). This was closely followed by those within 31–35 years with 69% increased odds of HBV infection (AOR: 1.69, 95% CI [1.20, 2.39]) and those within 36–40 years with 56% increased odds of HBV infection (AOR: 1.56, 95% CI [1.10, 2.24]) respectively. A p-value for trend within the age category of participants was significant (p = 0.029). As regards parity, compared with the nulliparous women, women with 3–4 had 32% (AOR: 0.68, 95% CI [0.50, 0.92]) decreased odds of HBV infection. A p-value for trend within the parity category was significant (p = 0.023). For the partner’s HIV status, compared with women with HIV-negative partners, those with HIV positive partners had a 53% increased odds of HBV infection (AOR: 1.53, 95% CI [1.22, 1.91]) and those with no partners had 46% increased odds of HBV infection (AOR: 1.46, 95% CI [1.13, 1.89]). The level of education, marital status and current ART regimen were not significantly associated with HBV infection in this study. None of our hypotheses were supported by the results.

As a by-product of the multiple imputation, FMI for age, partner’s age and parity were 0.012, 0.209 and 0.0019 respectively. FMI quantifies the amount of variable measures lost to non-response [30]. A sensitivity analysis comparing results obtained from list-wise deletion approach and multiple imputation for continuous variables showed there were no significant differences, so we reported results from list-wise approach because it had a full complement of both continuous and categorical outcome variables.

After modelling missingness for each variable, those with missing variables in parity (p = 0.027), ART regimen (p = 0.015) and years on ART (p = 0.012) were more likely to have had HBV positive outcome compared with those included in the analysis. Hence, these variables did not meet the criteria for characterization as missing completely at random (MCAR). For all other variables, there were no significant differences between those with missing variables and observed data and because missingness is independent of the observed data, we classified them as MCAR [29, 30]. We proceeded to interpret data from variables that are not categorized as MCAR with a note of caution.

## Discussion

This study investigated the prevalence and determinants of HBV co-infection among women of reproductive age living with HIV in Benue State, North Central Nigeria. Our results show that HBV is hyperendemic in this population. In contrast to our study hypothesis, HBV was most prevalent among women with lower parity, those aged 26–30 years and women with partners living with HIV infection.

The prevalence of HBV-HIV co-infection in this study is considerably higher than figures reported from other studies in Nigeria and other African countries. These figures range from 4.2% in Lagos [31] to 5.5% in Enugu [32], in southwest and southeast Nigeria respectively. A study of HBV-HIV co-infection in South Africa reported a prevalence of 3.4% [33]. These differences could be explained by distinctions in the populations studied and dissimilarities in baseline national HBV vaccination coverage amongst other factors. As a country, Nigeria enacted HBV vaccination into its expanded program for immunization (EPI) in 2004 [34], much later than most of its fellow African countries, however, low vaccine awareness, haphazard availability and unaffordability continue to reduce its uptake [35]. WHO reported 58% coverage for surviving infants who received third dose of HBV vaccine following birth dose in 2018 for Nigeria [36]. Our findings highlight the need for universal immunization of all newborns for HBV and for the early administration of hepatitis B immunoglobulin to infants of HBV-infected mothers.

Although different from our study’s context, we reviewed HBV prevalence estimates of other women of reproductive age not living with HIV from other regions in Nigeria and other African countries. The prevalence of HBV infection among HIV-uninfected women of reproductive age also varied in different regions of Nigeria, ranging from 7.3% [37] in the southwest to 12.3% [13] in the northwest regions of Nigeria. A meta-analysis of HBV infection in Nigeria also reported a prevalence of 14.1% among pregnant women attending ante-natal clinics [38]. However in other studies among women of reproductive age from other African countries they reported HBV prevalence of 3.7% in Rwanda [11], 3.8% in Botswana [10], 7.8% in Ethiopia [20] and 8% in Tanzania [9]. The differences noted between the HBV prevalence from Nigeria and other African countries might be similar to those observed in reproductive women living with HIV.

Previous studies [11, 20, 21] have described factors associated with higher odds of HBV infection among women of reproductive age. Younger age, lower level of education, higher previous number of children (parity), previous blood transfusion and history of induced abortions seemed recurrently associated with higher odds of HBV infection among women of reproductive age in Sub-Saharan Africa. Consistent with previous studies [11, 20, 21], this study found age and parity were associated with HBV infection. However, we found that having an HIV-positive partner or no partner was significantly associated with higher odds HBV infection among women. HBV and HIV have shared modes of transmission [39], but in these settings, it is difficult to ascertain the chronology of infection in concordantly infected couples. Studies [40, 41] have suggested that males have a higher prevalence of HBV compared with females in Nigeria. This may be a consequence of the non-participation of men in their wives or partner’s health program, a paradigm this study initiative is trying to shift among Nigerian men from grassroots [22]. Regarding parity of women, it was somewhat surprising that nulliparous women had the highest odds of HBV infection in our study contrary to previous studies [13, 15] which reported increased odds of HBV infection with increasing parity. It is difficult to explain this particular result; however, one possible explanation might be that lower parity HIV positive women are engaging in more unprotected sex with their HBV positive husbands to become pregnant. Although beyond the scope of this study, explanatory factors underlying this parity finding should be examined in follow-up research.

Consistent with other studies [11, 15, 21, 42], we found an association between age and HBV infection but there have been inconsistencies in the age group with the highest odds of HBV infection. In this study, the age group 26–30 years had the highest odds of HBV infection. Similar findings were reported by two other studies in Nigeria [15, 42]. On the contrary, a study in Rwanda [11] reported a seven-fold increased odds of HBV infection among younger women compared to the age group 26–30 years. In contrast, another study in Ethiopia reported highest odds of HBV infection among women older than 40 years [21]. These conflicting findings may be due to different cultures in the different contexts. For example, some African cultures [43–45] promote marriage at an early age (this exposes women to a higher odds of HBV infection for longer periods of their lives compared to those that married at more advanced age) while some promote marriage at older ages. Another possible explanation may be the lack of, or inadequate coverage of HBV vaccination for older women while they were younger, especially since most African countries did not include anti-HBV vaccines in their routine vaccination program until early 1990s [34]. Most of the women born before this era will be at least in their thirties (older age category) as at the time of this study. These findings have important implications for developing context-specific HBV prevention programs that are feasible and culturally acceptable to this population.

Our study has a few limitations. As with most cross-sectional studies, we are constrained to report associations and not causalities because it is difficult to ascertain temporality with these outcomes. Although this is a large cohort, the generalizability of this study to the Nigerian women might be challenging based on the diverse socio-demographic characteristics across the country. For example, most of the participants in this study were farmers; this is unlikely in a more urbanized setting. Previous studies [9, 12] reported employment as significant factors but we had limited variation in our employment categories because of our study setting. There is also a possibility of bias resulting from variable missingness. In most cases, the direction of this bias in relation to the outcome is unpredictable [46]. We, however, did a sensitivity analysis to get a clearer possible impact of this and we have suggested caution with the interpretation of results from the most affected variables (parity, ART regimen and duration on ART). Due to resource limitations, we did not measure hepatitis B surface antibody (anti-HBs), hepatitis B core antibody (anti-HBc) and hepatitis B Virus Deoxyribonucleic Acid (HBV-DNA) in this study.

Notwithstanding these limitations, this study is one of the largest cohorts in Nigeria that has investigated the prevalence and factors associated with HBV infection. Our findings contribute in several ways to a broader understanding of the unique trend of HBV characteristics in this population of women of reproductive age living with HIV. The findings from this study provide insights for hypothesis generation for further studies on HBV in this population and in low-resource health settings and setting health policies related to vaccination, screening and treatments for women. Examples of hypothesis for further studies will be to clarify the association between HBV status of women in reproductive age and their partner’s HIV’s status as well as parity of the women. There is an urgent need for the development and implementation of safe, feasible and innovative policies and interventions to address this scourge. Specific programs targeting HBV testing for all women of reproductive age group irrespective of their HIV status should be prioritized. In addition, we recommend a universal HBV vaccination for those that are HBV negative and treatment for those that are HBV positive on presenting for antenatal care.
